# Cancer Diaspora of Undifferentiated Cancer

**DOI:** 10.7759/cureus.52798

**Published:** 2024-01-23

**Authors:** Yuko Harada, Masao Toji

**Affiliations:** 1 Cardiology, Kawasaki Municipal Ida Hospital, Kawasaki, JPN; 2 Otolaryngology, Shin-Yurigaoka General Hospital, Kawasaki, JPN

**Keywords:** malignant melanoma, cancer diaspora, cancer of unknown primary site, laryngeal cancer, undifferentiated cancer

## Abstract

Undifferentiated cancer is a rapidly progressing cancer with poor prognosis. Sometimes, it is diagnosed at an advanced stage, and its origin is difficult to detect. A very unusual cancer was revealed by autopsy. The patient was an 83-year-old survivor of colon cancer, melanoma, and laryngeal cancer. He had been under watchful course observation after survival from laryngeal cancer but suddenly died due to aspiration pneumonia. The autopsy revealed undifferentiated cancer infiltrated the entire body, which was misdiagnosed with positron emission tomography (PET)/CT scan and MRI. The origin of this cancer was a mystery even with vigorous pathological evaluation. The patient was told that his previous cancers were all healed; however, undifferentiated cancer progressed rapidly to the entire body, just like “cancer diaspora”. This report highlights the limit of diagnostic imaging tools for aggressive cancer, sounding the alarm for clinicians to look beyond old presumptions.

## Introduction

Undifferentiated carcinoma is defined by the World Health Organization (WHO) as malignant epithelial neoplasms that lack evidence of glandular, squamous, or urothelial cell differentiation (differentiation refers to how histologically similar a neoplasm resembles normal tissues) [1]. Therefore, it is sometimes difficult to determine the origin at an advanced stage. Such cancer is called cancer of unknown primary site (CUP). CUP is a well-recognized clinical disorder, accounting for 3-5% of all malignant epithelial tumors. It is clinically characterized as an aggressive disease with early dissemination [2]. If a patient does not go to a hospital, the diagnosis of cancer will be delayed, and the origin of the advanced cancer cannot be detected. Then, can CUP occur in a patient who is watchfully monitored for cancer recurrence?

Here, we present a case of undifferentiated CUP with an aggressive clinical course that was unable to be diagnosed until autopsy, i.e., “cancer diaspora” that was impossible to predict yet fatal. The patient was a survivor of multiple cancers and was monitored for recurrence over decades. Even though he was admitted to a hospital for his last two months, undifferentiated CUP was not diagnosed by his physicians. The mystery was not solved even with vigorous pathological examinations.

Written consent for publishing was obtained from the patient’s family.

## Case presentation

An 83-year-old Caucasian man with a recent history of empyema and a past history of multiple cancers presented to an emergency department with respiratory failure. It took four hours for the ambulance to deliver the patient to a hospital because 66 hospitals refused to accept an ambulance due to the coronavirus disease 2019 (COVID-19) pandemic. The patient’s condition worsened in the ambulance. On arrival, oxygen saturation (SpO_2_) was only 80% with a full dose of oxygen (15 L/min). The patient presented with remarkable wheezing and rales due to purulent sputum. Blood tests revealed elevated WBC counts, kidney dysfunction, liver dysfunction, and elevated CRP levels as shown in Table 1.

**Table 1:** Blood test results on admission PLT: platelets; BUN: blood urea nitrogen; Cr: creatinine; eGFR: estimated glomerular filtration rate; UA: urinanalysis; AST: aspartate transaminase

Blood Cell Counts		Biochemistry				Blood Gas Analysis	
WBC	20,600/μl	BUN	41.0 mg/dl	ALT	131 IU/l	pH	7.24
Hb	9.6 g/dl	Cr	2.45 mg/dl	ALP	153 IU/l	pCO2	33.9 mmHg
PLT	41.0x104/μl	eGFR	20.5 ml/min/l	LDH	283 IU/l	pO2	66 mmHg
Neutrophils	96.80%	UA	8.0 mg/dl	Γ-GTP	18 IU/l	HCO3-	14.0 mmol/l
Eosinophils	0.00%	Na	133 mEq/l	CPK	33 U/l	BE	-12 mmol/l
Basophils	0.10%	K	5.3 mEq/l	CRP	19.54 mg/dl		
Lymph	2.30%	TB	0.4 mg/dl	BNP	61.6 pg/ml		
Monocytes	0.80%	AST	114 IU/l	PCT	2.58 ng/ml		

**Figure 1:**
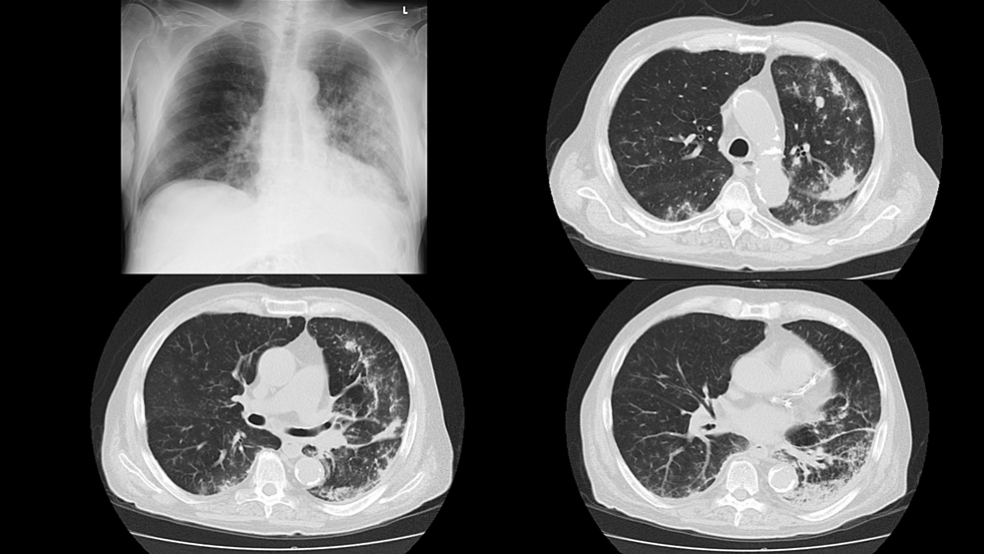
Chest X-ray and chest CT scan on admission Left top: chest X-ray reveals infiltration on the left lung. Right top: upper level of chest CT scan. Left bottom: middle level of chest CT scan. Right bottom: lower level of chest CT scan These CT scans reveal infiltrative shadows on both lungs, mainly on the left and dorsal sites.

Chest X-ray and a computed tomography (CT) scan demonstrated infiltration in both lungs as shown in Figure 1.

Given the diagnosis of bacterial pneumonia and multiple organ failure (MOF), treatment with antibiotics was initiated. However, the patient developed CO_2_ narcosis and went into cardiopulmonary arrest. After intubation, mechanical ventilation and noradrenaline were started. However, the patient did not recover from shock and died.

The cause of death was considered to be aspiration pneumonia because the patient had a recent history of empyema probably caused by mis-swallowing. However, the patient had been informed by a previous hospital that his prior pneumonia was completely cured upon discharge one week before his death. At that time, the patient was only informed that he had new bone metastasis of laryngeal cancer in his lumbar.

An autopsy was performed upon agreement of the patient’s family, which revealed undifferentiated cancer diffusely infiltrated in the lungs, liver, and subcutaneous tissue. The lumbar metastasis that was previously noticed also turned out to be undifferentiated cancer. Both lungs were infiltrated by undifferentiated cancer cells, and the bronchi were filled with purulent sputum. A bacterial culture of sputum revealed oral cavity resident bacteria, which supported the diagnosis of aspiration pneumonia. The cause of death was sepsis caused by aspiration pneumonia that was negatively affected by rapidly progressing systemic cancer.

It appeared to be a mystery where such a devastating and rapidly progressing cancer emanated from. The patient’s death occurred only one week after hospital discharge. The patient was told his pneumonia was completely cured. The patient was eating and drinking normally until his final day. His family did not notice any sign of mis-swallowing. The patient’s past history was thoroughly reviewed to solve the mystery.

Patient's history

**Figure 2:**
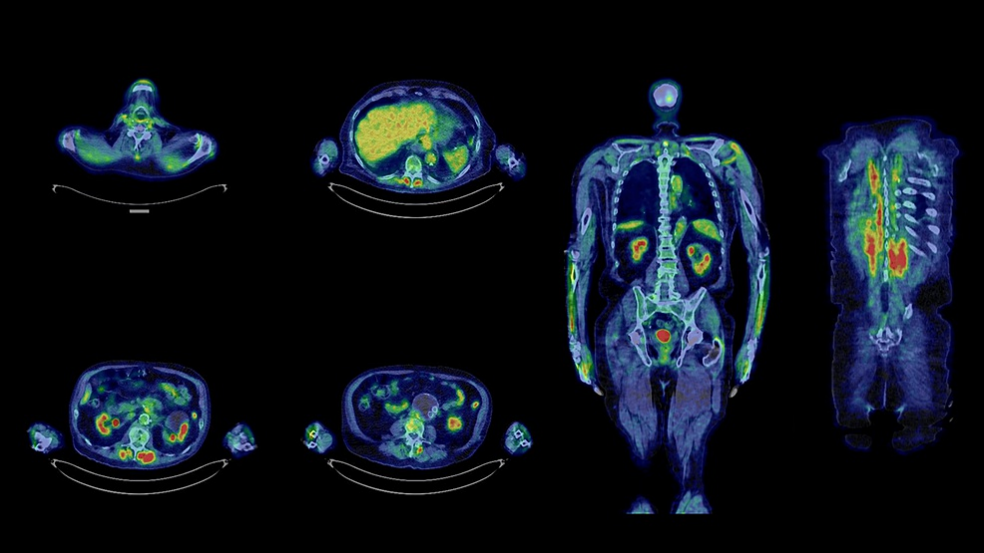
PET/CT scan after CyberKnife treatment Left: axial views. Right: coronal views FDG uptake is not observed in the throat. FDG uptake in the liver, kidneys, intestine, bladder, back muscles, and forearms was considered to be physiological. PET: positron emission tomography; FDG: fluorodeoxyglucose

The patient was diagnosed with skin cancer (possibly melanoma) in his 40s in the U.S. and underwent surgery in his back. After the resection of the cancer, a hole in the patient’s back remained for a while, which was filled by plastic surgery. The patient was an ex-smoker (20 cigarettes/day x 50 years), a daily alcohol drinker (3 glasses of wine/day), overweight, and hypertensive, otherwise his health was mostly fair. The patient was diagnosed with colon adenocarcinoma that was successfully resected by endoscopic surgery in his 70s. At the age of 75, the patient was diagnosed with melanoma in his left arm and underwent surgery. In the same year, he was diagnosed with laryngeal cancer (moderately differentiated squamous cell carcinoma, cT3N0) and underwent bioradiotherapy (BRT) with cetuximab and 70Gy radiation therapy. Eighteen months prior to death, he was diagnosed with a localized relapse of laryngeal cancer in mediastinal lymph nodes, however, a biopsy was deferred due to the COVID pandemic. Nivolumab was started but was discontinued due to its side effects such as severe fatigue, hypothyroidism, and interstitial pneumonia. CyberKnife (Accuray, California, USA) radiosurgery was performed and the tumors disappeared on a PET/CT scan as shown in Figure 2.

Even though the cancer was eradicated, severe fatigue remained. Six months after CyberKnife treatment, the patient developed aspiration pneumonia. Ten months after CyberKnife, the patient developed empyema that was treated with antibiotics (tazobactam/piperacillin, sulbactam/ampicillin, and meropenem) and chest drainage. Bacterial culture of blood and pleural effusion were negative. As the patient showed prolonged remittent fever and an elevated CRP level of 11.9 mg/dL, a whole-body CT scan with contrast medium was performed to search for the focus of inflammation or hidden cancer. CT scan revealed osteolytic changes in C5, C7, L3, and L4 of the spine, but the lungs were almost normal except for mild ground-glass opacity in the right middle lobe and a small nodule in the left upper lobe. Spine MRI and bone scintigraphy revealed that L3 and L4 had bone metastasis of cancer. The patient was discharged from the hospital because he did not exhibit difficulty in eating and walking, but he reported fatigue and mild fever. The patient had been informed that his fever was due to bone metastasis, and a bone biopsy was performed just before hospital discharge. However, the patient’s family noticed a small hump in the patient’s back near L3, and a skin biopsy was performed six days after hospital discharge. Pathological evaluation was difficult for both biopsy samples. Metastatic poorly differentiated carcinoma was suspected; however, the origin was unknown. The patient developed respiratory failure eight days after hospital discharge and died within 23 hours thereafter.

Autopsy and pathological examination

The results of the autopsy are listed in Table 2.

**Table 2:** Results of the autopsy

Organ	Detected cancer	Details
Left vocal cord	None	Origin of laryngeal cancer, post chemotherapy and radiation therapy
Left lung	Undifferentiated	Weighted 840 g; 9 mm nodule in upper lobe; diffuse cancer invasion to pleura and vessels
Right lung	Undifferentiated	Weighted 1325 g; diffuse cancer invasion to pleura and vessels
Trachea, bronchi	None	Filled with content of stomach; bronchopneumonia; Gram-positive coccus (+)
Liver	Undifferentiated	Weighted 940 g; diffuse cancer invasion to vessels
Vertebra	Undifferentiated	3 cm cancer mass protruding from L4
Skin, subcutaneous tissue	Undifferentiated	Cancer invasion mainly in subcutaneous tissue from the upper to lower back
Lymph nodes	Undifferentiated	Cancer invasion in the neck and hilar nodes
Left upper arm	None	Origin of melanoma, post-surgery
Prostate	Adenocarcinoma	12 mm latent cancer; Gleason score 3+4=7, EPE0, ly0, v0, pn1, sv0, pT2a, pN0
Abdominal aortic aneurysm	None	95 x 80 x 55 mm; with stent inserted; without rupture

**Figure 3:**
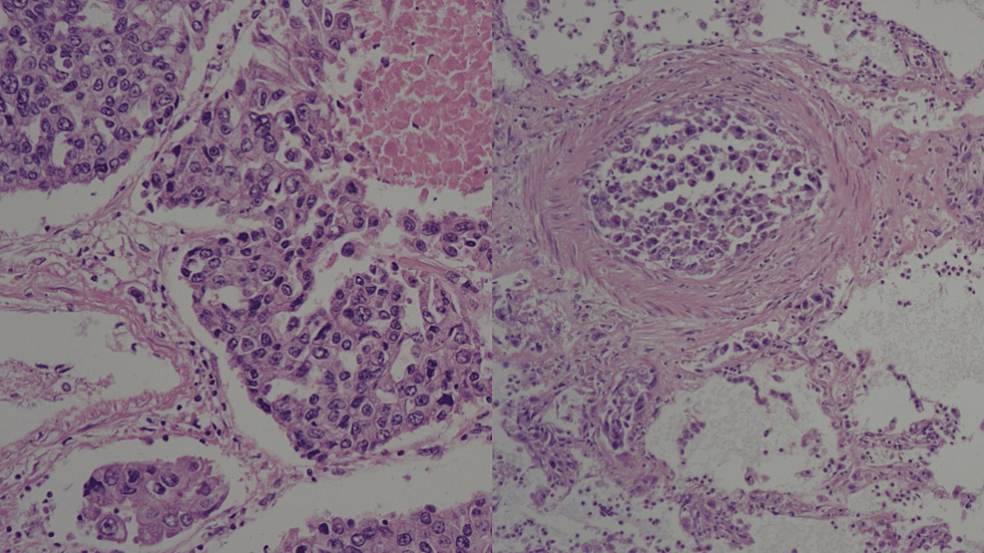
Cancer cells in the lungs Left: the lung was infiltrated with cancer cells. Right: the vessels in the lung were filled with cancer cells.

The lungs and the liver were almost completely infiltrated with cancer cells (Figure 3).

**Figure 4:**
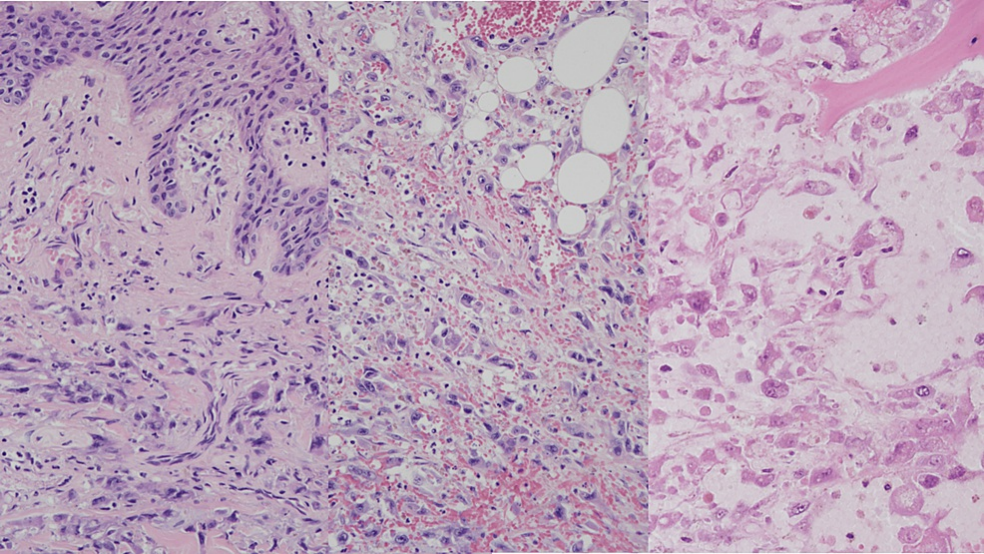
Cancer cells infiltrated in the skin and the spine Left: cancer cells infiltrated in the skin. Middle: cancer cells infiltrated in subcutaneous tissue. Right: cancer cells infiltrated in L3.

The blood vessels and lymph vessels of these organs were filled with cancer cells. The pleura, spine (L3 and L4), and back skin at the level of T4 and L3 were also infiltrated with cancer cells (Figure 4). All the specimens demonstrated the same cancer cells that were morphologically poorly differentiated or undifferentiated carcinoma. The origin of the cancer was undetectable.

**Figure 5:**
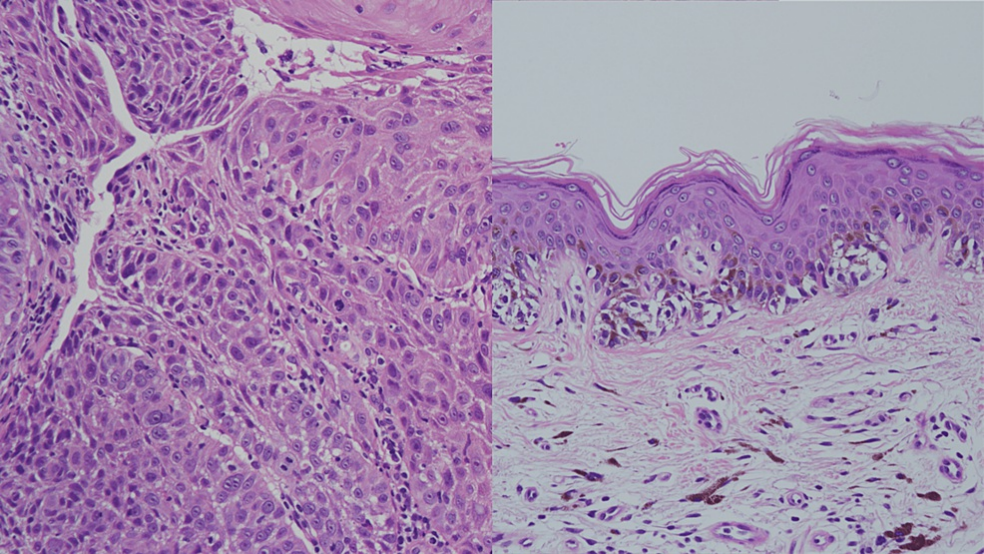
Surgery samples of previous cancers Left: Biopsy sample of previous laryngeal cancer seven years ago showing moderately differentiated squamous cell carcinoma. Right: Surgery sample of melanoma eight years ago showing infiltration of melanocytes.

According to the patient’s past history, the candidates of cancer origin were laryngeal cancer and melanoma. The post-surgery scar of previous melanoma in the patient’s back was also infiltrated with cancer cells. A biopsy sample of the previous laryngeal cancer seven years ago was ordered to compare with the current cancer, which showed moderately differentiated squamous cell carcinoma (Figure 5). The surgery sample of previous melanoma was also ordered to compare with the current cancer, which showed infiltration of melanocytes (Figure 5). Therefore, the current cancer was proven to have a different morphology than previous laryngeal cancer or melanoma. It was therefore not possible to detect cancer origin by comparing surgical samples of previous cancers.

**Figure 6:**
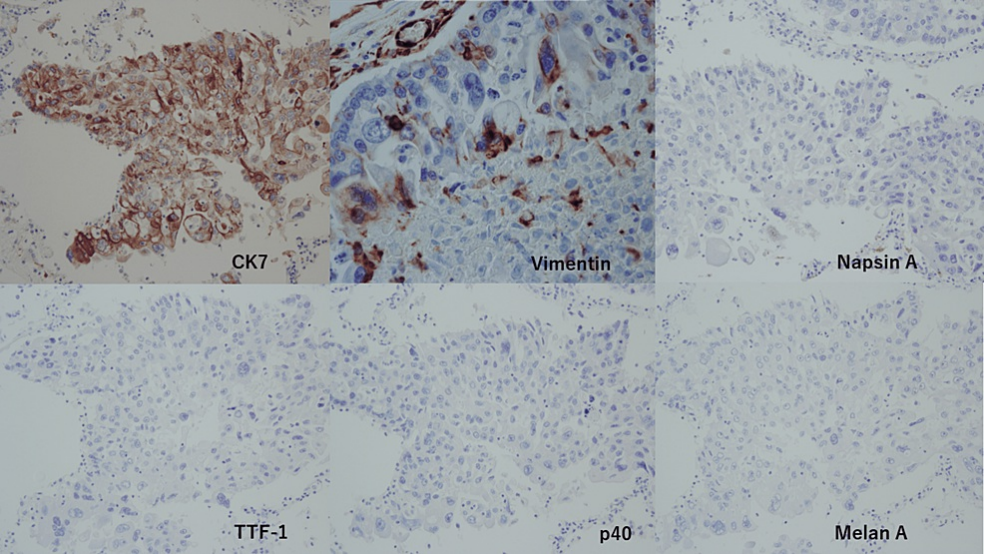
Histopathological examinations of the cancer cells in the lungs Top from the left: CK7 (+), vimentin (+), and Napsin A (-). Bottom from the left: TTF-1 (-), p40 (-), and Melan A (-).

Next, a histopathological examination was performed to elucidate whether the cancer cells were derived from squamous cell carcinoma, melanoma, or elsewhere. A pertinent immunohistochemical panel was performed; AE1/AE3 (+), p40(-), CK7 (+), CK20 (-), Napsin A (-), TTF1 (-), S100 (-), SOX10 (-), Melan-A (-), HMB45 (-), and vimentin (+) (Figure 6). These results did not match either squamous cell carcinoma or melanoma. The histomorphological and immunohistochemical features favored the diagnosis of undifferentiated cancer of unknown primary.

To evaluate if the cancer was mutated from previous laryngeal cancer, the previous sample from eight years ago was ordered to perform a gene examination. However, the sample amount was not enough for a full evaluation. OncomineTM Dx Target Test (Thermo Fisher Scientific, Inc., Massachusetts, USA), a next-generation sequencing assay designed to detect variants in multiple genes associated with cancer, was performed for previous laryngeal cancer and the sample from the autopsy. EGFR、ALK, ROS1, and BRAF genes were all negative for both samples. The origin of the cancer remained unknown.

## Discussion

Without the COVID pandemic, the patient would have had a different clinical course. First, the lymph node biopsy would have been performed when the relapse in the mediastinum was first detected, as the lymph node swelling might have been the origin of the undifferentiated cancer that caused death. Second, a skin biopsy could have been performed earlier to diagnose undifferentiated cancer. Third, the ambulance might have been able to deliver the patient to a hospital quicker to save his life. No such tragedy should be repeated.

The origin of the cancer remained unknown even after immunohistopathology and gene examination. The OncomineTM DX Target Test was the only gene testing available in the hospital. Further evaluation was deferred due to a lack of amount of samples from previous laryngeal cancer. This is a limitation of this study.

Metastatic adenocarcinoma is the most common histopathology (80%) for cancer of unknown primary (CUP) [2]. In head and neck cancer, squamous cell carcinoma is the most common (90%) for CUP, and the others are adenocarcinoma, melanoma, and undifferentiated carcinoma [3,4]. Undifferentiated cancer is rare; therefore, the prevalence of undifferentiated CUP is not well defined. A review from Germany reported that the histology of CUP comprises adenocarcinoma (50% to 70%), undifferentiated carcinomas (20% to 30%), and squamous cell carcinomas (5% to 8%) [5]. It is natural that undifferentiated cancer is revealed as CUP because it progresses rapidly. There are no statistics of undifferentiated CUP in Japan due to its rarity [6].

CUP has a poor prognosis. In the Swedish cancer registry of 18,911 CUP patients, patients presenting with affected inguinal lymph nodes had a median survival of 18 months, and patients affected intra-abdominally had a median survival of only four months [7]. A retrospective analysis of 223 patients of CUP (adenocarcinoma or undifferentiated carcinoma) reported that the median survival from the time of diagnosis was 16.5 months [8]. The said patient died 18 months after diagnosis with localized relapse of laryngeal cancer in mediastinal lymph nodes, which could have almost been the natural course as CUP. However, the said patient would have survived a little longer if the ambulance had been able to deliver him to a hospital quicker.

The concept of “cancer diaspora” was advocated by Professor Kenneth J. Pienta in 2013 [9]. “Diaspora” means the movement, migration, or scattering of people away from an established homeland. Professor Pienta applied the concept of "diaspora" to cancer metastasis, saying “a diaspora is dispersed, usually expelled, from a single origin” [9]. The said patient’s cancer behaved just the same as “cancer diaspora”. The origin was unknown, but the cancer spread very quickly all over his body probably within a few months.

Immune checkpoint inhibitors, such as anti-PD-1 (programmed cell death 1)/PD-L1 (programmed cell death 1-ligand 1) are well-known cancer drugs, however, they may cause anecdotal rapid progression of cancer. Champiat S et al. reported that an aggressive pattern of hyperprogression exists in a fraction of patients treated with anti-PD-1/PD-L1 [10]. The patient was treated with nivolumab, which is anti-PD-1 18 months prior to his death, however, it is difficult to prove whether nivolumab caused the progress of cancer or not.

It is not known what triggered such dispersion of cancer. It is also not known whether the undifferentiated cancer was mutated from squamous cell carcinoma in the larynx. It may even have been nivolumab or CyberKnife radiation therapy that triggered the dispersion of cancer, or it might even have occurred naturally. Whichever it is, cancer diaspora may occur in spite of watchful observation with CT and MRI.

## Conclusions

A rare case of devastating undifferentiated carcinoma in multiple cancer survivors is described. Even with watchful observation over a period of years, this cancer was still not detected with current diagnostic imaging tools. Such “cancer diaspora” may occur and clinicians should be alerted.

If it were not for the COVID-19 pandemic, this patient may have survived a little longer. However, the undifferentiated cancer was drastically progressive to cause malaise, leading to fatal aspiration pneumonia. Two months of in-hospital care with repeated blood tests and CT scans failed to detect "cancer diaspora."
